# Jiao-Tai-Wan Ameliorates Depressive-Like Behavior through the A_1_R Pathway in Ovariectomized Mice after Unpredictable Chronic Stress

**DOI:** 10.1155/2020/1507561

**Published:** 2020-09-18

**Authors:** Lina Xiang, Yuan Feng, Qianqian Hu, Jiahui Zhu, Ren Ye, Zhengzhong Yuan

**Affiliations:** ^1^Wenzhou Medical University First Affiliated Hospital, Wenzhou, Zhejiang Province 325000, China; ^2^Wujiang District Hospital of Traditional Chinese Medicine, Suzhou City, Suzhou, Jiangsu Province 215000, China; ^3^Wenzhou Hospital of TCM Affiliated to Zhejiang Chinese Medicine University, Wenzhou, 310053 Zhejiang Province, China; ^4^Wenzhou Medical University, Wenzhou, Zhejiang Province 325000, China; ^5^Department of Traditional Chinese Medicine, Wenzhou Medical University First Affiliated Hospital, Wenzhou, Zhejiang Province 325000, China

## Abstract

**Objective:**

This study was aimed at observing the effect Jiao-Tai-Wan in menopausal depression.

**Methods:**

In this paper, we used ovariectomized mice subjected to chronic unpredictable stress as a menopausal depression model. After the chronic stress, mice were administrated with JTW (3.3 and 6.6mg/kg) and imipramine (10 mg/kg) for 14 days. On the 14th day, mice were subjected to the behavior test like the forced swim test, tail suspension test, and locomotor activity or were sacrificed to assess the protein changes in different brain regions.

**Results:**

The administration of JTW at doses of 3.3 and 6.6mg/kg (p.o.) significantly shortened the duration of immobility in forced swim and tail suspension tests. There was no obvious difference in locomotor activity among all the groups. The western blot analysis data indicated that treatment with JTW (3.3 and 6.6 mg/kg, p.o.) prominently increased the A_1_R protein and the downstream protein ERK1/2 levels in the prefrontal cortex and hippocampus. However, the administration of JTW did not influence c-Fos protein in either the prefrontal cortex or hippocampus.

**Conclusion:**

Our findings suggest that JTW plays a vital role in ameliorating menopausal depression symptoms in the A_1_R-ERK1/2 pathway in the prefrontal cortex and hippocampus.

## 1. Introduction

The relevance of gender differences in major depressive disorder (MDD) is well-known: the risk in women is twice as high as that in men [1], especially during menopause. Menopause is an inevitable phase in women's life, and it can cause a series of physical problems, such as hot flashes, headache, insomnia, and mood swings [2], which may affect quality of life. Evidence indicates that hormone fluctuations, but not absolute estradiol levels, are considered to be involved in depression and that the use of exogenous estrogen might mitigate depressive symptoms in perimenopausal women [3]. Although estrogen primarily modulates the level of serotonin [4], which is a key neurotransmitter in depression, the onset of side effects has been of concern in menopausal women receiving this therapy [5]. In addition, classic antidepressant drugs, such as tricyclic antidepressants (imipramine), have serious side effects, such as nausea, vomiting, a decreased level of consciousness, and tachycardia [6]. Hence, the discovery of safe and effective pharmacotherapy to improve this symptom in women is urgently needed.

Jiao-Tai-Wan (JTW) is a well-known prescription used in patients with many kinds of disease for a long time. JTW is composed of *Coptis chinensis* (CC) and cinnamon (CIN) [7], both of which can lower neurological inflammation and ameliorate the behavior of cognitive dysfunction [8, 9]. Increasing evidence has pointed out that cognitive function is a measure of the progression of depression [10]. Additionally, a study elucidated that JTW played a vital role in alleviating depression-like behavior through the monoaminergic response and the anti-inflammation pathway [11]. Therefore, we can speculate that JTW may have the power to improve depressive symptoms in menopausal women. However, information on this activity of JTW in menopausal women is still unknown.

To date, researchers have found that major depression is related to improved adenosine A_1_ receptor (A_1_R), extracellular signal-regulated kinase 1/2- (ERK1/2-), and c-Fos expression [12–14]. Adenosine, as a neuromodulator, is the metabolite of ATP production, which is important for affecting synaptic transmission and neuronal excitability in the central nervous system (CNS) [15]. Some studies have indicated that adenosine receptors could be prime candidate targets in the regulation of cognitive processes, sleep intention, and major depression amelioration [16–18]. In the A_1_R transgenic mouse model, Serchov et al.'s study showed that enhanced A_1_R levels have antidepressant effects in a depression-like model [12]. A 10-year follow-up study found that caffeine, an adenosine nonselective antagonist, can reduce the risk of depression in women but not men [19]. Considering the abovementioned findings, we wondered whether JTW would show antidepressant-like effects by regulating the concentration of A_1_R, which is rarely studied. In the present research, we also investigated the effect of JTW on regulating the concentration of the popular proteins ERK1/2 and c-Fos in depressive-like ovariectomized mice.

## 2. Materials and Methods

### 2.1. Animals

Female 6-week-old outbred ICR mice weighing 20-22 g were obtained from the Animal Center of Shanghai Branch, Chinese Academy of Sciences. The mice were housed five per cage under standard colony conditions, with controlled ambient temperature (22 ± 1°C), humidity (50 ± 10%), and a 12 h natural light/dark cycle. Mice had free access to food and water and were allowed to acclimate for 5 days prior to the experiment. All procedures were performed in compliance with the National Institutes of Health Guide for Care and Use of Laboratory Animals (Publication No. 85–23, revised 1985), as approved by the Wenzhou Medical College Committee on current ethical regulation for Animals Care and Use.

### 2.2. Drug and Drug Administration

The main ingredients of Jiao-Tai-Wan are Coptidis rhizome and cinnamon, which were extracted three times in a ratio of 10 : 1 by boiling and digesting, and solid particles were kept. The solid particles of Jiao-Tai Wan were diluted with distilled water to a final concentration of 3.3 mg/kg and 6.6 mg/kg, according to the mass ratio of human to mouse, and given orally (p.o.). Imipramine is a positive drug, purchased from Hunan Dongting Pharmaceutical Co., Ltd. (Hunan, PR China), was diluted to 10 mg/kg with distilled water, and administered intraperitoneally (i.p.). In this paper, JTW (3.3 and 6.6 mg/kg) and imipramine (10 mg/kg) were administered daily for 14 days. The behavioral testing commenced 60 min after the last drug treatment.

### 2.3. Surgery

At the beginning of the experiment, mice were ovariectomized. Ovariectomy was performed under pentobarbital sodium (50 mg/kg, i.p.) anesthesia. Surgery was performed after mice showed a reduced respiratory rate and blunted responses when the foot pad was softly pinched. A midventral incision was made, the oviducts were then ligated, and the bilateral ovaries and ovarian fat were removed. The sham groups were subjected to the same surgical procedure except for the removal of the ovaries. The surgical incision was closed with sutures, and the mice were allowed to recover for 1 week with daily observed postsurgical recuperation.

### 2.4. Forced Swim Test (FST)

The forced swim test was conducted according to the procedure that has been described previously [20], with minor modifications. Briefly, the mice were individually subjected to a pretest for 15 min in glass containers (height: 25 cm, diameter: 10 cm) containing water up to 19 cm at 24 ± 1°C. After 24 h, mice were again placed in the same system for a period of 6 min (out of which 2 min was for habituation). The duration of immobility was recorded during the last 4 min of the test by two independent observers blinded to the experiment. Immobility was defined as the mice floating motionless in the water and ceasing struggling, while making only small movements necessary to keep the head above the water.

### 2.5. Tail Suspension Test (TST)

The tail suspension test was based on the procedure that has been described previously [20], with minor modifications. Mice were individually suspended 50 cm above the floor by adhesive tape (approximately 1 cm from the tip of the tail). The duration of immobility was determined during the final 4 min of the 6-min testing period by two independent observers blinded to the experiment. Immobility behavior was defined as the mice remaining completely motionless.

### 2.6. Locomotor Activity

Locomotor activity was measured by electronic counters in five activity chambers (DigBehav, Jiliang Co., Ltd., Shanghai, China), with a minor modification [20]. When the paws of mice contacted or disconnected from active beams, which were in a random configuration, beam breaks were converted into pulses, which were proportional to the locomotor activity of mice and were automatically kept as the cumulative total counts of motor activity. Each mouse was placed in the chambers for 15 min and allowed to acclimatize for 5 min. Then, locomotion counts were recorded for a period of 10 min.

### 2.7. Chronic Unpredictable Stress Procedure

The mice were subjected to the chronic unpredictable stress protocol developed by [21, 22], with minor modifications. Mice were exposed to 2 different stressors twice daily between 8:00 am and 16:00 pm over a period of 14 consecutive days. The order of the stressors used was as Table 1.

On day 14 (60 min after the drug or vehicle administration), mice were used for experiments including the forced swim test (FST), tail suspension test, and locomotor activity or were sacrificed to assess the protein changes in different brain regions.

### 2.8. Western Blot Analysis

Mice were decapitated, and the hippocampus and prefrontal cortex were rapidly dissected and stored at −70°C. Tissue samples were homogenized in RIPA buffer supplemented with protease and phosphatase inhibitors and centrifuged at 13,000 rpm for 30 min at 4°C. The supernatant was quantified using a bicinchoninic acid assay kit (Beyotime Institute of Biotechnology Co., Ltd., Shanghai, China) for total protein concentrations. Samples were heated in a metal bath for 5 min, and after cooling down, the protein was loaded onto SDS-PAGE and transferred to polyvinylidene difluoride membranes. Membranes were then blocked with 5% fat-free milk for 1 h at room temperature and washed three times with Tris-buffered saline with 0.1% Tween 20 (TBST). Then, the samples were incubated with the appropriate primary antibodies overnight at 4°C (anti-c-Fos, 1 : 1000; anti-A_1_R, 1 : 1000; anti-ERK1/2, 1 : 1000; anti-vinculin, 1 : 1000). After washing, the membranes were incubated with a 1 : 10000 dilution of mice or rabbit peroxidase-conjugated secondary antibodies at room temperature for 1 h. After that, the membranes were washed with TBST three times. The detection quantification of particular proteins was determined with a ChemiScope (PowerPac™ Basic, Singapore) analysis program. All bands were standardized with the internal reference vinculin.

### 2.9. Statistical Analysis

All statistics were performed using GraphPad Prism 6 (GraphPad Software Inc., San Diego CA, USA). The values are presented as the means ± S.E.M. One-way analysis of variance (ANOVA) following a post hoc Dunnett test was used to determine the significant differences among groups. To compare two groups, data were analyzed by an unpaired *t*-test. A value of *p* < 0.05 was considered to be significant.

## 3. Results

### 3.1. Effects of JTW on the Duration of Immobility in the FST and TST in Perimenopausal Depression Model Mice

**Figure 1:**
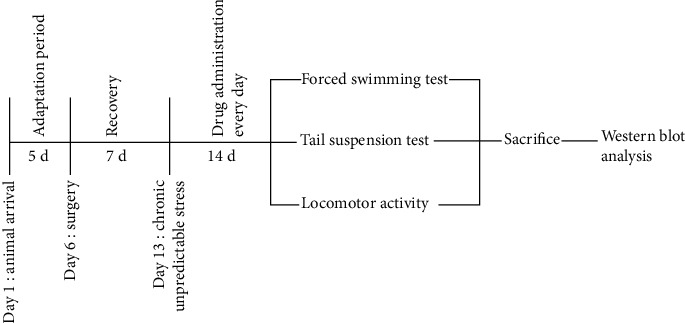
Experimental timeline for drug treatments. After surgery, mice were administrated with JTW for 14 days. All the behavioral tests were performed 24 h after last drug treatment; then, the mice were sacrificed and biochemical assays were performed.

**Figure 2:**
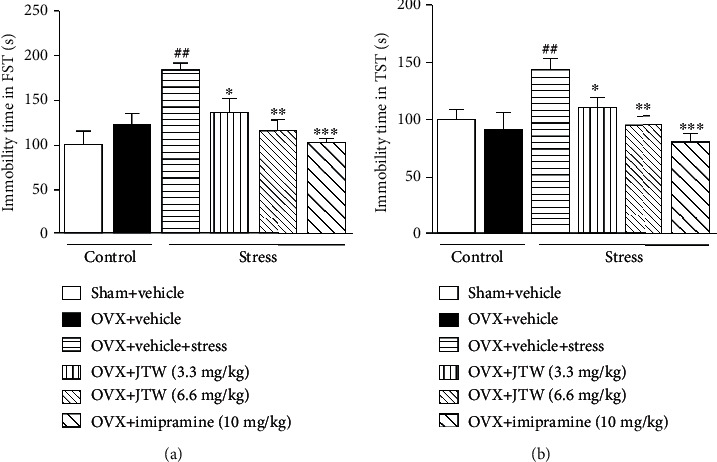
The effects of JTW on the duration of immobility in the forced swimming (a) and tail suspension (b) tests. Mice were administered vehicle, JTW (3.3 and 6.6 mg/kg) or imipramine (10 mg/kg) once daily for 14 consecutive days. On the last day, mice were subjected to the behavioral tests after 30 min drug administration. Values are the mean ± S.E.M. with 6 mice in each group. Compared with vehicle-treated ovariectomized mice, ^#^*p* < 0.05; compared with vehicle-treated ovariectomized mice subjected to chronic unpredictable stress, ^∗^*p* < 0.05 and ^∗∗^*p* < 0.01.

The experimental procedure is shown in Figure 1. We assessed the chronic effects of JTW on the immobility time of perimenopausal depression-like behavior in forced swim and tail suspension tests. In the ovariectomy groups, we did not observe any behavioral changes in the FST and TST compared to the behaviors of the vehicle-treated sham-operated group. As shown in Figure 2, exposure to chronic unpredictable stress significantly increased the duration of immobility in FST and TST in the vehicle-treated ovariectomized mice compared to vehicle-treated sham-operated mice (*p* < 0.01). The duration of immobility in the FST and TST was robustly and significantly altered after JTW (3.3 and 6.6 mg/kg, p.o.) was chronically administered for 2 weeks (*p* < 0.05). According to the results, the dose of 6.6 mg/kg was more effective than the lower dose (3.3 mg/kg, p.o.) in the FST and TST. All the results coincided with the results of the positive control drug imipramine (10 mg/kg, i.p.) in that immobility time was significantly reduced in both the FST and TST.

### 3.2. Effects of JTW on Locomotor Activity in Menopausal Depression Model Mice

**Figure 3:**
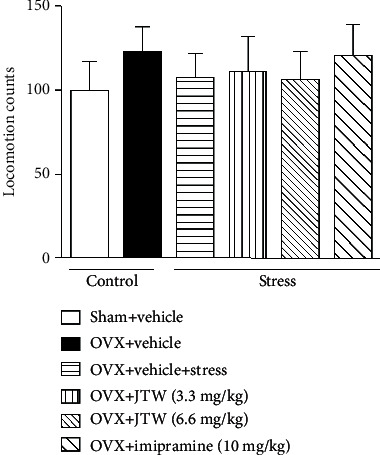
The effects of JTW on locomotor activity. Mice were administered vehicle, JTW (3.3 and 6.6 mg/kg), or imipramine (10 mg/kg) once daily for 14 consecutive days. On the last day, mice were subjected to the behavioral tests after 30 min drug administration. Locomotion counts were recorded for 10 min. Values are the mean ± S.E.M. with 6 mice in each group. There were no obvious differences compared with vehicle-treated ovariectomized mice subjected to chronic unpredictable stress.

To rule out the potential effects of JTW on overall movement in antidepressant-like behavior tests, mice were assessed in the open-field experiment to evaluate locomotor activity. As Figure 3 shows, the locomotor activity in vehicle-treated ovariectomized mice was not influenced compared to that in the vehicle-treated sham-operated group. Moreover, neither JTW (3.3 and 6.6 mg/kg, p.o.) nor imipramine (10 mg/kg, i.p.) significantly affected locomotion, with mice showing similar locomotor activity to that in the vehicle-treated sham-operated group. Both doses obviously decreased the duration of immobility in the FST and TST.

### 3.3. Effects of JTW on A_1_R Expression in the Prefrontal Cortex and Hippocampus

**Figure 4:**
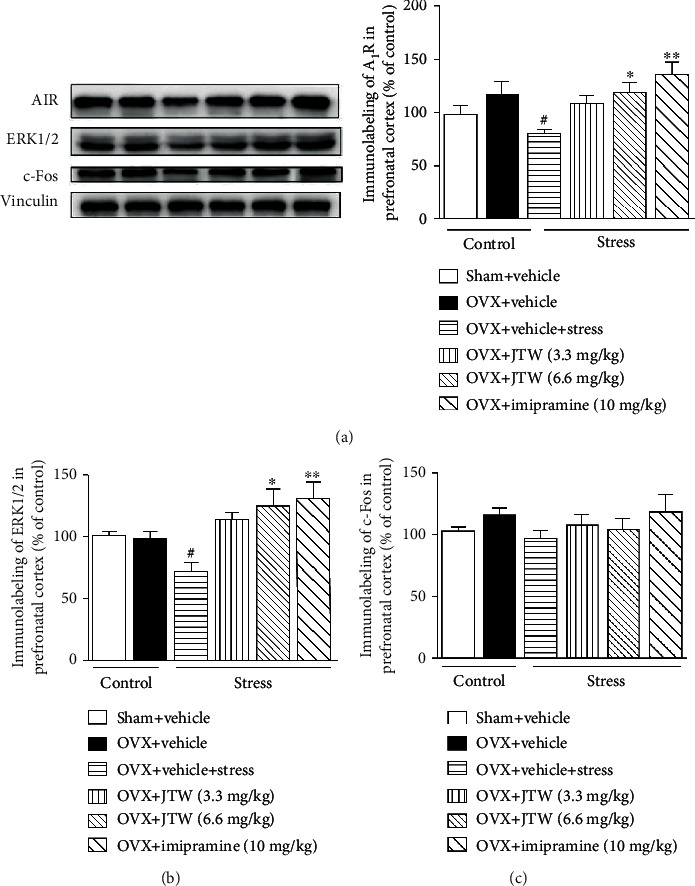
Effects of JTW on protein of A_1_R (a), ERK1/2 (b), and c-Fos (c) expression in the prefrontal cortex. Mice were administered vehicle, JTW (3.3 and 6.6 mg/kg), or imipramine (10 mg/kg) once daily for 14 consecutive days. On the last day, mice were sacrificed after 30 min drug administration. Values are the mean ± S.E.M. with 6 mice in each group. Compared with vehicle-treated ovariectomized mice, ^#^*p* < 0.05; compared with vehicle-treated ovariectomized mice subjected to chronic unpredictable stress, ^∗^*p* < 0.05 and ^∗∗^*p* < 0.01.

**Figure 5:**
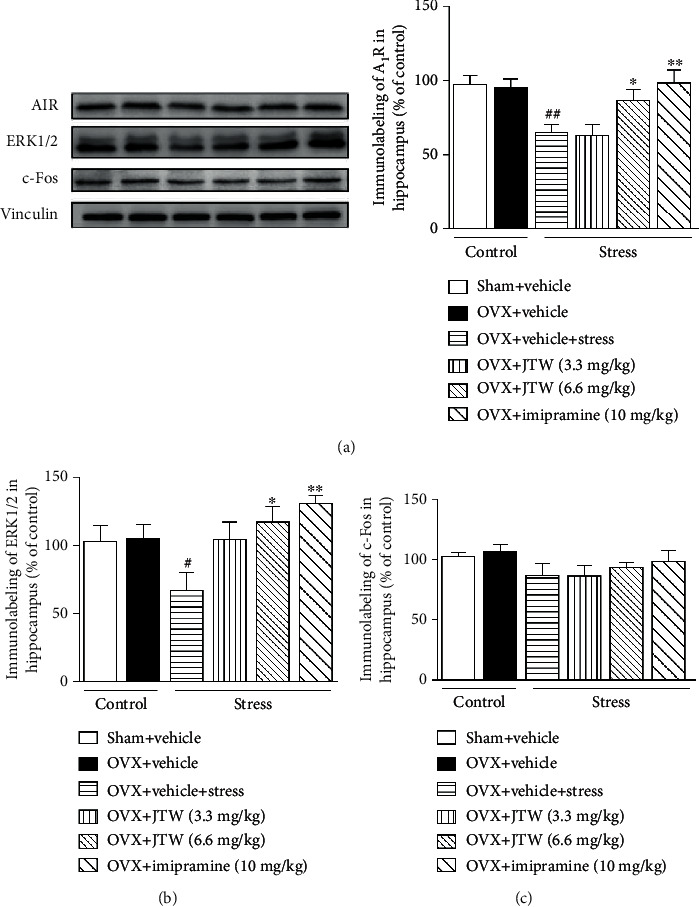
Effects of JTW on protein of A_1_R (a), ERK1/2 (b), and c-Fos (c) expression in the hippocampus. Mice were administered vehicle, JTW (3.3 and 6.6 mg/kg), or imipramine (10 mg/kg) once daily for 14 consecutive days. On the last day, mice were sacrificed after 30 min drug administration. Values are the mean ± S.E.M. with 6 mice in each group. Compared with vehicle-treated ovariectomized mice, ^#^*p* < 0.05; compared with vehicle-treated ovariectomized mice subjected to chronic unpredictable stress, ^∗^*p* < 0.05 and ^∗∗^*p* < 0.01.

To investigate whether the chronic administration of JTW changed the A_1_R expression in the prefrontal cortex and hippocampus, we performed a western blot experiment. As shown in Figures 4(a) and 5(a), A_1_R levels were remarkably downregulated in the prefrontal cortex and hippocampus in vehicle-treated ovariectomized mice subjected to chronic unpredictable stress compared to levels in the vehicle-treated sham-operated group (*p* < 0.05). After 2 weeks of treatment with JTW, the A_1_R expression was slightly higher after the administration of the 3.3 mg/kg dose, and this reduction returned to baseline levels with the 6.6 mg/kg dose (*p* < 0.05). The effects coincided with those of the positive control drug imipramine (*p* < 0.01).

### 3.4. Effects of JTW on ERK1/2 Expression in the Prefrontal Cortex and Hippocampus

The expression of the ERK1/2 protein was determined in the prefrontal cortex and hippocampus using representative western blot experiments. As illustrated in Figures 4(b) and 5(b), chronic stress induced an obvious reduction in ERK1/2 protein expression in vehicle-treated ovariectomized mice compared to levels in the vehicle-treated sham-operated group (*p* < 0.05) in the prefrontal cortex and hippocampus. This alteration was efficaciously prevented by the administration of JTW (3.3 and 6.6 mg/kg, p.o.) for 14 days (*p* < 0.05). Imipramine, as the positive control drug, confirmed our results.

### 3.5. Effects of JTW on c-Fos Expression in the Prefrontal Cortex and Hippocampus

Furthermore, we sought to determine whether treatment with JTW for 14 days would alter the expression of c-Fos in the prefrontal cortex and hippocampus. To our knowledge, c-Fos expression is related to neuronal activity [23]. As displayed in Figures 4(c) and 5(c), there were no significant differences between c-Fos levels in vehicle-treated ovariectomized mice subjected to chronic unpredictable stress compared to those in the vehicle-treated sham-operated group. Furthermore, the expression of c-Fos was not different in mice subjected to JTW versus imipramine treatment.

## 4. Discussion

Numerous population-based studies have shown that women in the transition to menopause are more vulnerable to depression. In a 6-year follow-up study of 460 women who had no previous history of depression, Cohen et al. revealed that those in the menopausal period were much more likely to suffer from obvious depressive symptoms than those who remained in the premenopausal period [24]. Also, in the Penn Ovarian Aging Study, the risk of depression during the menopausal transition was almost three times higher than that during the premenopause stage [25]. However, there are a few drugs directed against menopausal depressive states in middle age women that are presently used as clinical antidepressants. It is well-known that menopausal women with depressive symptoms usually use estrogen replacement treatment, but some women cannot tolerate the side effects, such as a greater risk of cancer incidence [20]. Additionally, evidence has indicated that women experiencing mood disorders in the menopausal phase could not tolerate combined hormone replacement therapy and treatment with antidepressants [26]. Thus, candidates naive to antidepressants who are able to ameliorate symptomatic menopausal depression by effective and secure character means must be considered.

Stressful incidents are pivotal factors for mood disorders. Unpredictable chronic mild stress, as a classic and acceptable model, was used in this paper, as this type of stress is similar to the daily stress experienced by human beings [27]. Research on depression found that animals showed depressive-like behavior after exposure to unpredictable chronic stress, which was ameliorated by traditional medicine such as *trans*-resveratrol or other types of antidepressants [28]. Diminishing levels of ovarian hormones not only influence reproductive function and sexual differentiation but are also related to psychiatric symptoms and learning memory abilities [29]. Numerous researchers have confirmed the model of bilateral ovariectomy as the classic method to investigate the pharmacological and toxicological mechanisms of menopausal depression [30]. In this paper, we performed bilateral ovariectomy surgery following unpredictable chronic mild stress in mice, as a model of menopausal depression, which was already performed by Ma and his/her coworkers on 2013 [31], to investigate the effect of JTW on menopausal depression symptoms and its mechanism. JTW consists of *Coptis chinensis* (CC) and cinnamon (CIN), which have been shown to be effective in ameliorating cognitive impairments by decreasing the level of neurological inflammation [32]. In contrast, a study of the Aphrodite capsule, which contains cinnamon, noted a statistically obvious alleviation of menopausal symptoms in 50–60-year-old postmenopausal women [2]. Recently, Zhe et al. revealed that JTW significantly changed observed behaviors in the TST and FST without inhibiting the exploration of the center in the OFT [11]. However, there is a lack of direct evidence on the effect of JTW on menopausal depression or on the molecular basis of its response in the brain. Our study showed that mice exposed to chronic unpredictable stress for 2 weeks following ovariectomy showed progressively longer durations immobile in the TST and FST, a behavior which was interestingly modified by the administration of JTW (3.3 mg/kg and 6.6 mg/kg), which regulated the concentration of A1R, ERK1/2 and c-Fos protein in the brain.

The adenosinergic system is popular and is able to modulate mood symptoms, playing an important role in different kinds of psychiatric diseases, such as depression [18] and anxiety [33]. To our knowledge, this is the first study to reveal that chronic treatment with JTW (6.6 mg/kg) significantly decreased the duration of immobile time in both the FST and TST, by enhancing the level of A_1_R in different brain regions (hippocampus and prefrontal cortex) in menopausal depression-like mice. Moreover, our results did not show obvious differences in the behavior experiments or biochemistry analysis between the OVX mice and the sham group that received vehicle after chronic stress. The inhibition of the central nervous system might also affect the change in immobility time in the TST and FST. To rule out this interference, we performed a locomotor test for further confirmation, and no difference in locomotion was found in any group. In line with our observation, A_1_R might occupy a vital position in the field of menopausal depression, especially in treatment with JTW, which may be accepted as an alternative medicine for women. Our results coincided with those of other studies that knocked out adenosine receptors or changed the concentration of adenosine and inosine, significantly showing depressive-like behavior in the FST and TST after chronic stress [34]. In the depression model of sleep deprivation, increased A_1_R levels could evoke an antidepressant effect [35]. In addition, the antidepressant effect of imipramine was consistent with enhanced A1R expression, which decreased the duration of immobile time in the depression model [12]. In this context, the adenosine A1 receptor is considered to be a promising target for protective or therapeutic treatment in depressive disorders.

Although the A1R pathway was found to be important in depression, we also considered ERK1/2 and c-Fos to be involved. In learning, memory, and neuroplasticity, ERK signaling channels have been studied extensively, which indicates that ERK1/2 changes after stress stimulation and antidepressant treatment [36]. Interestingly, there is a research that clarified that the homer1a induction is a crucial joint mechanism mediating the antidepressant effects in which pathway the protein of A1R was a key factor. Furthermore, the A_1_R agonist MRS5474 improved the level of ERK1/2 and hormer1a which implied that A_1_R have the ability to mediate the concentration of ERK1/2 [12]. This report corroborated our result that ERK1/2 was significantly enhanced in the hippocampus and prefrontal cortex in menopausal depression-like mice after treatment with JTW (6.6 mg/kg), due to the fact that there is no relevant reports that studied the change of A_1_R and ERK1/2 after treatment of Jiao-Tai-Wan in a menopausal depression mice model. Thus, in this paper, we examined alterations in ERK1/2 levels after treatment with JTW in menopausal depression-like mice. In our experiment, we also did run the western blotting of *p-*ERK1/2 for countless times with nothing that appeared. We tried different methods, and different experimenters came out with the same consequence. We were so confused with the result, so we decided to look into the relative proteins like Elk-1, ATF, Ap-1, c-Fos, and c-Jun which were mediated by the *p-*ERK1/2. As we know, A_1_R caused synaptic plasticity in neurons and so did the protein of c-Fos which has the ability to affect the plasticity-relative genes [37]. Then, we supposed that A_1_R might influence the concentration of c-Fos to improve the disease of depression through the intermediate substance ERK1/2/*p-*ERK1/2. As the result presented, there was nonresponsive c-Fos in the hippocampus and prefrontal cortex after the treatment of Jiao-Tai-Wan. So, we guess that the stimulation and treatment of Jiao-Tai-Wan could affect the concentration of ERK1/2 but are not strong enough or we did not seize the right time to see the change of *p-*ERK1/2 and c-Fos. According to the cited references, external stimulus type and strength can affect the expression of c-Fos, which might be a marker of nociception [38, 39]. It is essential to figure out the changes in *p*-ERK1/2 and c-Fos after JTW treatment in menopausal depression-like mice in our further work, and we will consummate the pathway.

Taken together, our results demonstrated that JTW has the same capabilities as antidepressants in the FST and TST, which may be due to changes in the levels of A_1_R and ERK1/2 proteins. This is the first paper to investigate the function of JTW in menopausal depression-like mice and the involvement of the A_1_R protein. Additionally, *p*-ERK1/2 and c-Fos protein may be affected, if we can predominate the appropriate stress intensity and best timing before it is metabolized. These findings are particularly relevant considering that they may open a new pathway to better explain the mechanism of the effect of JTW on depression. Further studies are focused on synaptic plasticity after the treatment of JTW and complete the pathway involved in A_1_R, ERK1/2, *p*-ERK1/2, and the downstream protein like c-Fos which maybe related with different timing in menopausal depression-like mice.

## Figures and Tables

**Table 1:** 

Days	1	2	3	4	5	6	7
Stressors	Food deprivation (24 h)	Water deprivation (24 h)	Cold swim (10°C, 5 min)	Shaking (high speed, 45 min)	Food deprivation (24 h)	Tail pinch (1 min)	Water deprivation (24 h)
	Restraint (4 h)	Tail pinch (1 min)	Wet sawdust (4 h)	Lights on overnight (6 h)	Social isolation (6 h)	Cold swim (6°C, 8 min)	Shaking (high speed, 1 h)
Days	8	9	10	11	12	13	14
Stressors	Foot shock (30 min; 1 mA, 1 s duration average 1 shock/min)	Food deprivation (24 h)	Lights on overnight (12 h)	Tail pinch (1 min)	Water deprivation (24 h)	Foot shock (45 min; 1 mA, 1 s duration average 1 shock/min)	Food deprivation (24 h)
	Restraint (6 h)	Social isolation (24 h)	Wet sawdust (6 h)	Cold swim (4°C, 10 min)	Switching cages (8 h)	Shaking (high speed, 1.5 h)	Wet sawdust (8 h)

## Data Availability

All data generated or analyzed during this study are included in this article.
